# End-of-life decisions and practices as viewed by health professionals in pediatric critical care: A European survey study

**DOI:** 10.3389/fped.2022.1067860

**Published:** 2023-01-10

**Authors:** Anna Zanin, Joe Brierley, Jos M. Latour, Orsola Gawronski

**Affiliations:** ^1^ Department of Women's and Children's Health, University of Padua, Padua, Italy; ^2^ Critical Care Units, Great Ormond Street Hospital, London, United Kingdom; ^3^ School of Nursing and Midwifery, University of Plymouth, Plymouth, United Kingdom; ^4^ Professional Development, Continuing Education and Research Service, Bambino Gesù Children's Hospital, IRCCS, Rome, Italy

**Keywords:** end of life, pediatric critical care, decision making, ethics, end of life (EOL), decision making

## Abstract

**Background and Aim:**

End-of-Life (EOL) decision-making in paediatric critical care can be complex and heterogeneous, reflecting national culture and law as well as the relative resources provided for healthcare. This study aimed to identify similarities and differences in the experiences and attitudes of European paediatric intensive care doctors, nurses and allied health professionals about end-of-life decision-making and care.

**Methods:**

This was a cross-sectional observational study in which we distributed an electronic survey to the European Society of Paediatric and Neonatal Intensive Care (ESPNIC) members by email and social media. The survey had three sections: (i) 16 items about attitudes to EOL care, (ii) 14 items about EOL decisions, and (iii) 18 items about EOL care in practice. We used a 5-point Likert scale and performed descriptive statistical analysis.

**Results:**

Overall, 198 questionnaires were completed by physicians (62%), nurses (34%) and allied health professionals (4%). Nurses reported less active involvement in decision-making processes than doctors (64% vs. 95%; *p* < 0.001). As viewed by the child and family, the child's expected future quality of life was recognised as one of the most critical considerations in EOL decision-making. Sub-analysis of Northern, Central and Southern European regions revealed differences in the optimal timing of EOL decisions. Most respondents (*n* = 179; 90%) supported discussing organ donation with parents during EOL planning. In the sub-region analysis, differences were observed in the provision of deep sedation and nutritional support during EOL care.

**Conclusions:**

This study has shown similar attitudes and experiences of EOL care among paediatric critical care professionals within European regions, but differences persist between European regions. Nurses are less involved in EOL decision-making than physicians. Further research should identify the key cultural, religious, legal and resource differences underlying these discrepancies. We recommend multi-professional ethics education to improve EOL care in European Paediatric Intensive Care.

## Introduction

Most childhood deaths occur in hospitals (1–5), with around 70% occurring in paediatric intensive care units (PICU) (1, 3, 4), most following the withdrawal or withholding of life-sustaining treatment (3–6). PIC teams must, therefore, anticipate, identify and effectively treat dying children's pain and suffering and provide psychosocial and spiritual support to each child and their family (4).

There is growing international literature describing attitudes and barriers to delivering optimal EOL care in PIC (6–9). A combination of recent demographic changes and public and professional attitudes means that in some high-income countries, most children admitted to PIC have a life-limiting condition and represent a significant proportion of PIC deaths (10). Some studies have described the successful integration of paediatric palliative care teams into PIC to support the family and the care team. But their expertise is traditionally in non-PIC settings (9–12), and delivering EOL in PIC remains the responsibility of the intensive care team.

How care is delivered to a dying child carries significant consequences for all involved. Several studies outline the importance of providing grief and bereavement support to both the patient-family unit and the healthcare professionals (HCPs) involved. Such support is vital to allow healthcare professionals (HCP) to continue to provide ongoing high-quality EOL care (13–16). The international Paediatric Intensive Care Unit Model of Integrated Care study reported global disparities in PIC grief and bereavement care provision (16). Despite individual national reports (6, 8, 14), there is no broad overview of contemporary EOL practice in European PIC though one study, performed over a decade ago, did investigate decision-making in the forgoing of life-sustaining therapy by PIC physicians and nurses (17).

One emerging concern is the different perceptions that nurses and physicians have about the objectives and goals of EOL decision-making (18, 19). Such disagreement between PIC nurses and physicians has been identified as a significant obstacle in delivering high-quality EOL care (20).

How PIC EOL decisions are made has important consequences on the quality and effectiveness of the child's care, the team's relationship with the family, the hospital team's functioning, and the long-term well-being of the PIC workforce (21). The last is increasingly recognised as vital given the increasing workforce pressures and prevalence of moral distress, fatigue and burnout reported in PIC teams (16, 21, 22). In addition, PIC-HCPs face novel challenges in delivering recurrent EOL care with the expanding use of prolonged technological support, such as ECMO, and innovative attempts at cure (23). There is an increasing focus on PIC teams' resilience and situational self-awareness to ensure they can continue to deliver care (16), with individual or collective interventions including relevant education processes or debriefs (21).

To date, few studies have investigated the decision-making process and attitudes of PIC physicians and nurses towards EOL care in Europe (18, 19). One concern to explore at the outset is the crucial observation that nurses and physicians have different perceptions about the objectives and processes of EOL decision-making (20, 24). Furthermore, disagreements about EOL planning among nurses create a significant obstacle to delivering high-quality care (25). Therefore, this study aimed to identify similarities and differences in the experiences and attitudes of European PIC doctors, nurses and allied health professionals regarding EOL decision-making and practices.

## Methods

The End-of-life Views Of heaLth professionals in paediatric critical carE (EVOLvE Study) is a cross-sectional observational study using a survey design performed between January and October 2019. We use the Checklist for Reporting Results of Internet E-Surveys (CHERRIES) (26) to report the study. We conducted this study in accordance with the principles of the Declaration of Helsinki (Brazil 2013) and the General Data Protection Regulation (E.U. 2016/679). The European Society of Paediatric and Neonatal Intensive Care (ESPNIC) scientific committee reviewed and approved the study.

### Instrument

The questionnaire consisted of 48 items (Supplementary Material S1). It was translated from English by bi-lingual clinicians into three European languages (English, French, and Spanish) using a recognised cultural adaptation process (27) and tested by local clinicians for face validity.

### Participants and recruitment

At the launch of the questionnaire, the total ESPNIC membership was 709 individual members: 565 doctors and 144 nurses and allied health professionals (AHPs). All ESPNIC members received an email invitation to complete the online questionnaire. We also disseminated the questionnaire through social media (Facebook and Twitter) and sent a reminder email to ESPNIC members after one month. In addition, the questionnaire was available on the online platform SurveyMonkey. Participants signed an electronic informed consent form when completing the survey, and the data were anonymised. There was no compensation or reimbursement for participation in the EVOLvE Study.

### Analysis

Data was collected and recorded without identifiers, protected by Secure Sockets Layer (SSL) encryption, and analysed in aggregate form. We excluded respondents from outside Europe, those working entirely in NICU settings and uncompleted questionnaires (2 items missed or more). We used IBM SPSS Statistics 25 for Windows for the statistical analysis and performed descriptive statistical analysis, presenting categorical data as *n* (%) and using student's *t*-tests, chi-square tests and ANOVA where appropriate. Significance was set at <0.05.

We conducted subgroup analysis for professional profiles and country distribution, with distribution for northern, central and southern European countries adopted from the Ethicus Study. In Ethicus, countries were classified into three European regions (28); Northern Europe (Ireland, Latvia, Lithuania, the Netherlands, Sweden, Finland and the United Kingdom), Central Europe (Austria, Belgium, Germany, France, Luxembourg, Poland, and Switzerland), and Southern Europe (Bulgaria, Italy, Portugal, Spain, Greece, Turkey). We performed multivariate statistical analysis using correspondence analyses and summarised the analysis of the two-way contingency table in which the observed association between the region and the responses to the different options. The inference in correspondence analysis is whether certain levels of each region are associated with the degree of agreement to one item. Correspondence analyses produces a graphical representation of the data. On each axis, the percentage of inertia shows the variance of the plot explained by the principal component.

## Results

**Figure 1:**
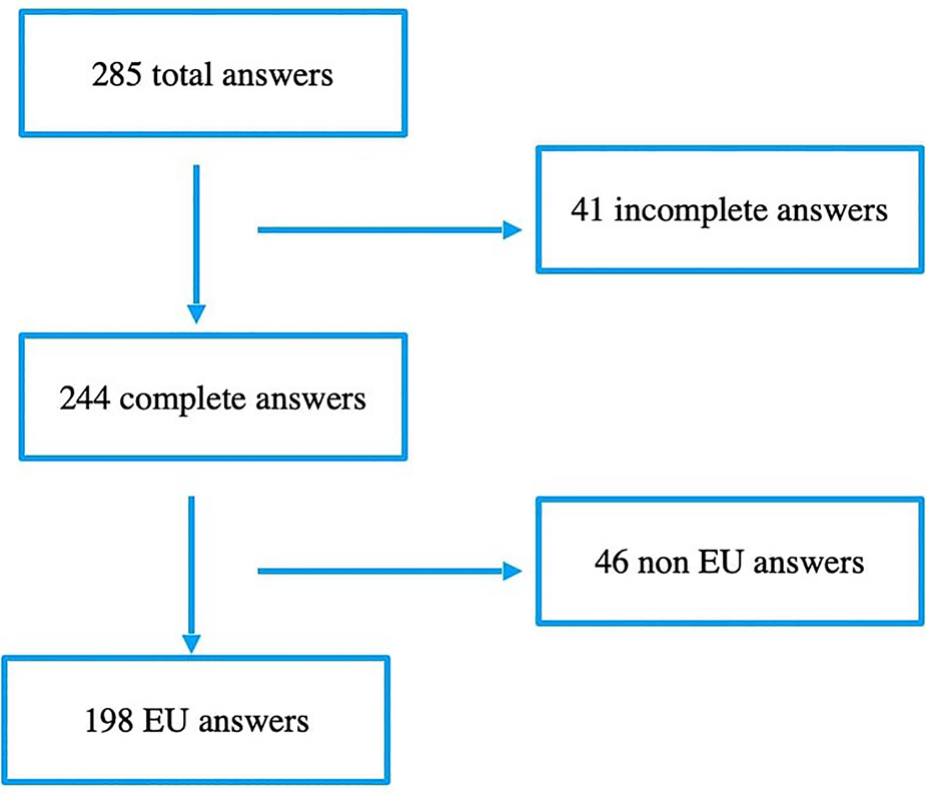
Flowchart of the study. EU (European).

**Figure 2:**
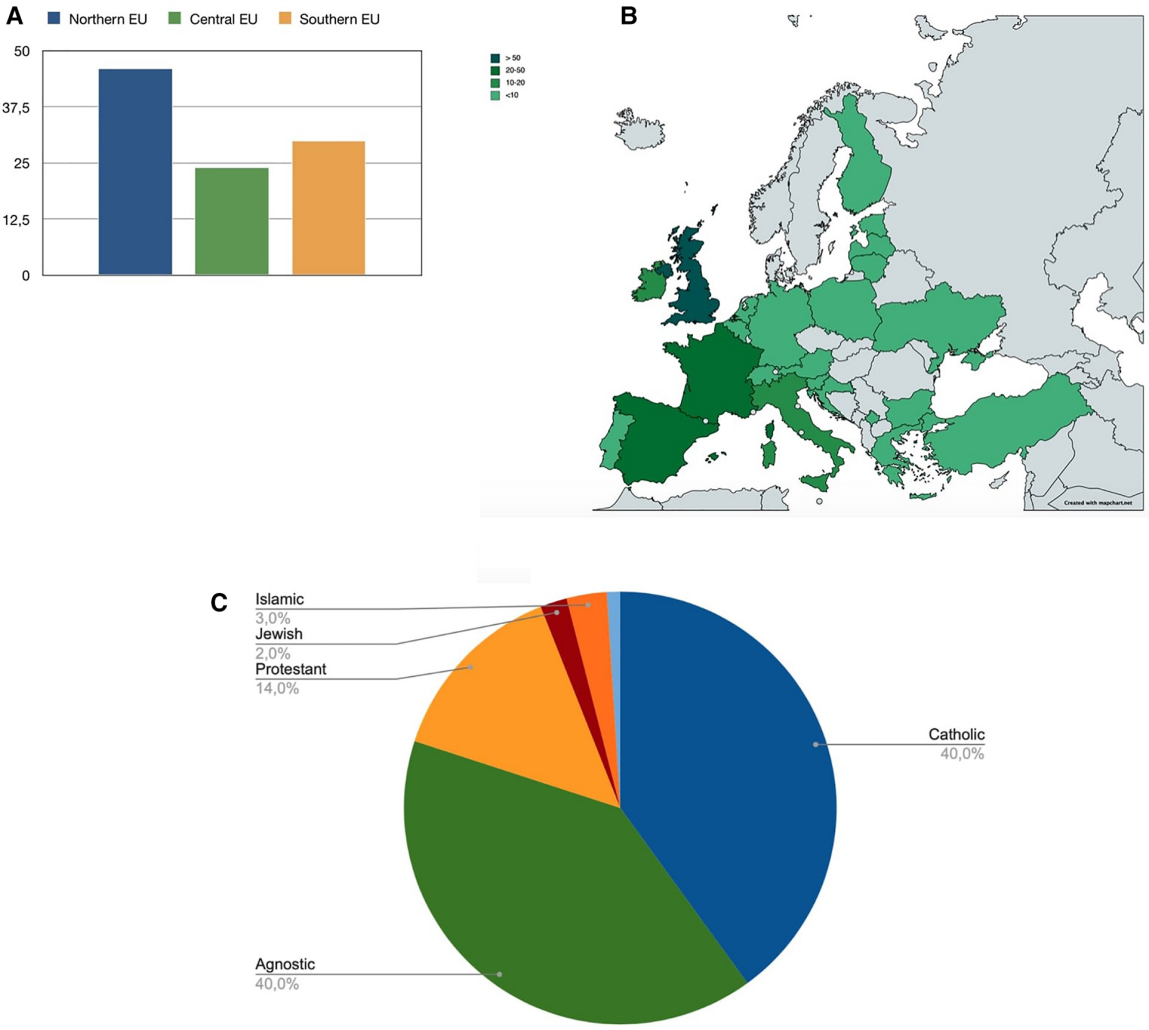
Distribution of respondents by European region (%, figure **A**) and country (N, figure **B**). EU (Europe). (**C**) Distribution of respondents by religious background.

From January to October 2019, we collected 285 responses (Figure 1). All items were completed in 244 questionnaires, of which 198 were by European HCPs and therefore included in the final analysis. The majority of respondents were female (*n* = 145; 73%), physicians (*n* = 120; 62%), and working in PIC (*n* = 158; 80%). We present respondents' characteristics in Table 1, showing the countries most represented to be the United Kingdom (*n* = 64, 32%), Spain (*n* = 34, 17%) and France (*n* = 32, 16%) and region, country and religious background in Figures 2A–C.

**Table 1:** Demographic characteristics of the cohort of European respondents.

Respondents' characteristics	Total = 198
**Gender**	
Female, *n* (%)	145 (73%)
**Age**	
Age, median (range)	40 (23–66)
Age, mean (DS)	40,9 (9,8)
Year in intensive care, median (range)	10,5 (0–37)
Year in intensive care, mean (DS)	12,9 (9,2)
**Work setting**	
Combined PICU/NICU (%)	22 (11%)
NICU (%)	18 (9%)
PICU (%)	158 (80%)
**Role**	
Physician	120 (62%)
Nurse	71 (34%)
Allied Health professional	7 (4%)
**Religious background**	
Agnostic or atheist (%)	80 (40%)
Catholic (%)	79 (40%)
Protestant (%)	28 (14%)
Islamic (%)	6 (3%)
Jewish (%)	3 (2%)
Buddhist (%)	2 (1%)
**European region**	
Northern EU countries	91 (46%)
Central EU countries	48 (24%)
Southern EU countries	59 (30%)

EU, European; NICU, neonatal intensive care unit; PICU, pediatric intensive care unit. For subgroup analysis, the Europe countries were classified into three European regions as in the ETHICUS study (9); northern (Ireland, Latvia, Lithuania, the Netherlands, Sweden, Finland and United Kingdom), central (Austria, Belgium, Germany, France, Luxembourg, Poland, and Switzerland), and southern (Bulgaria, Italy, Portugal, Spain, Greece, Turkey). *Results are expressed as number (%) and median (25th–75th percentiles).

**Figure 3:**
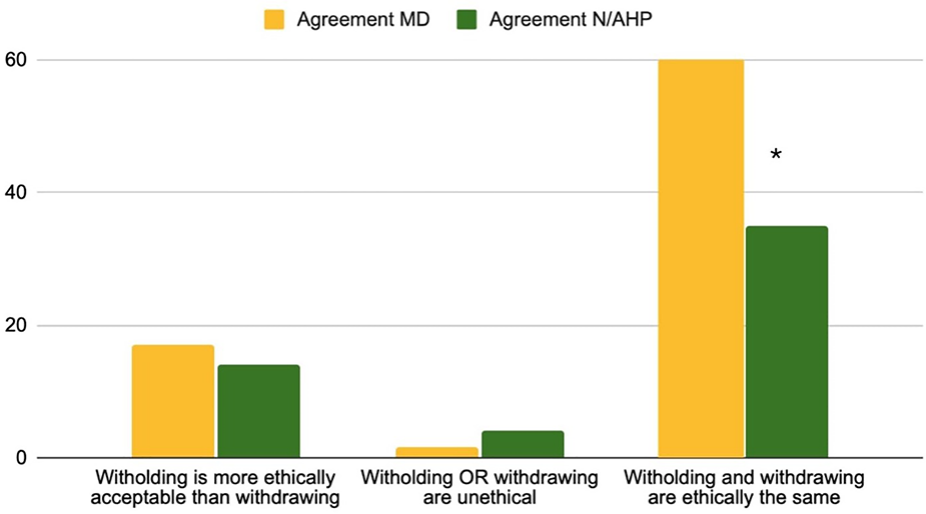
Question 1–3 statements about withholding and withdrawing life-supporting treatments % of agreement MD = physicians, N/AHP = nurses and allied health professionals.

**Figure 4:**
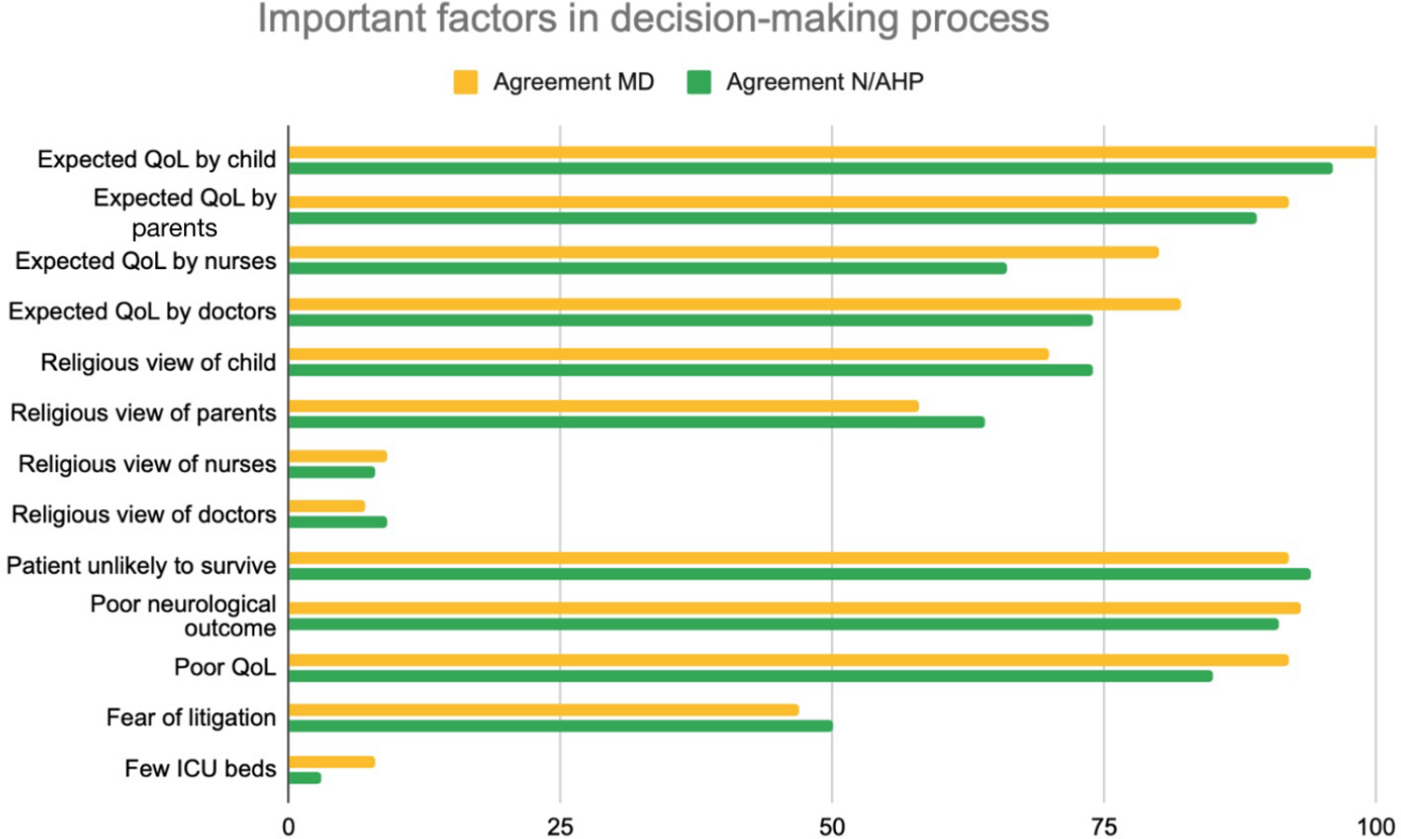
Question 4–16 attitudes towards end of life care: important factors in the decision-making process. % of agreement for MD = physicians, N/AHP = nurses and allied health professionals. QoL (Quality of Life), ICU (Intensive Care Unit).

The first part of the questionnaire focused on statements about withholding and withdrawing life-sustaining treatments. The distribution of the answers for all 48 items is reported in Supplementary Material S1. The view that “withholding and withdrawing are ethically the same” was distributed equally, i.e., agreement and disagreement with the sentence was balanced. However, a significant difference occurred in professional subgroup analysis, with nurses and AHP disagreeing that these approaches are ethically equivalent (Figure 3). No main differences were identified in considering essential factors in EOL decisions about withholding or withdrawing life-sustaining treatment. Expected quality of life of the child, likelihood of survival and poor neurological outcome were the main factors reported as important by healthcare professionals. Religious views of both families and the healthcare team was considered as least important (Figure 4).

**Figure 5:**
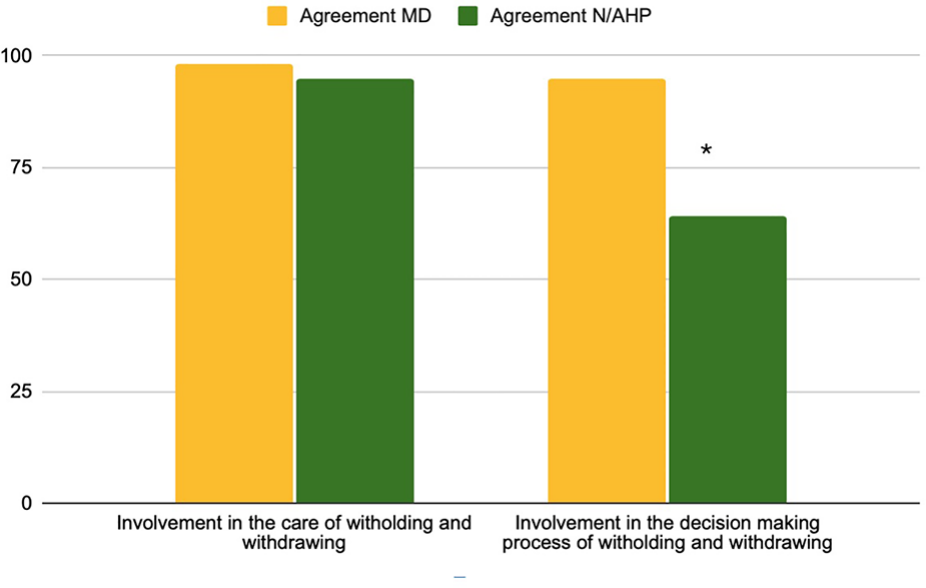
Questions 17–18 involvement in care and decision-making process % of the agreement for MD = physicians, N/AHP = nurses and allied health professionals.

**Figure 6:**
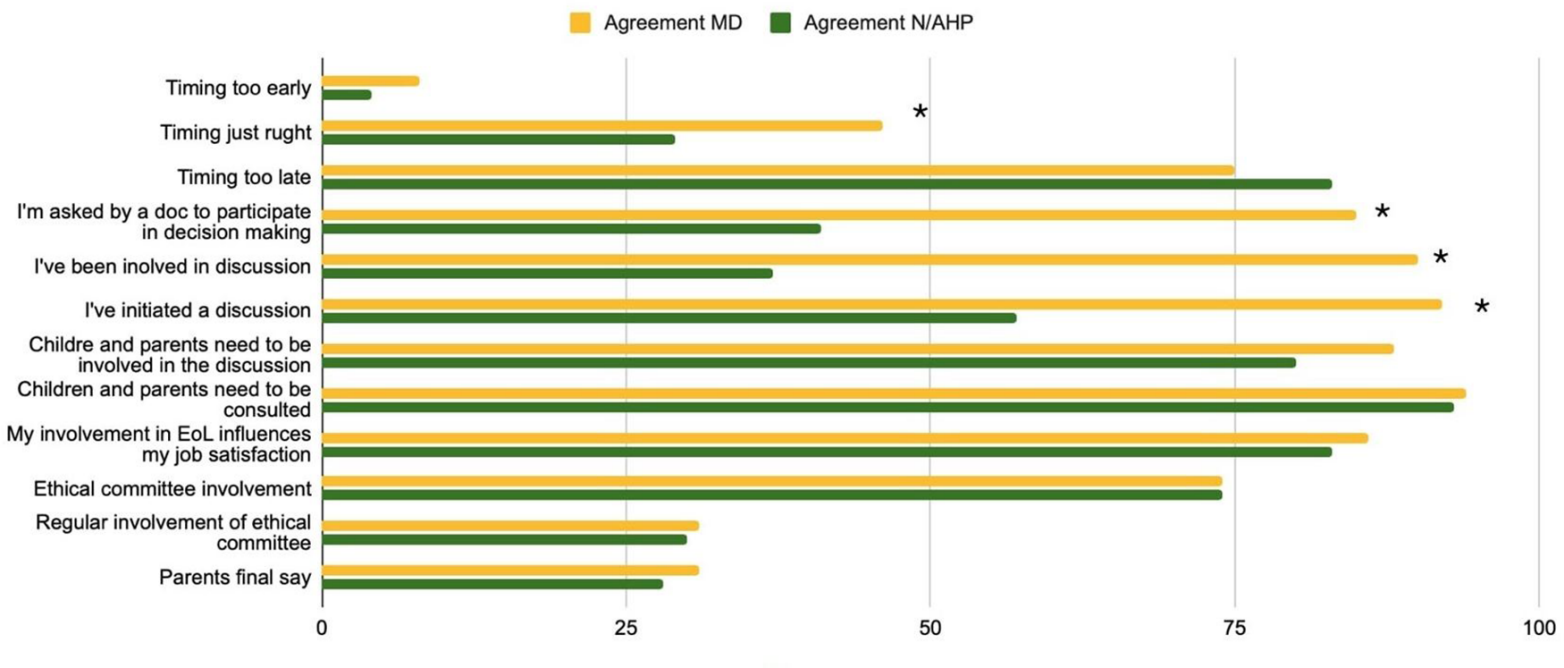
Questions 19–30. Involvement in End of Life (EoL) decision % of the agreement for MD = physicians, N/AHP = nurses and allied health professionals.

In the second section of the questionnaire about involvement in EOL decisions, high involvement by all respondents was perceived in both the delivery of care (*n* = 191; 97% Supplementary Material) and in the decision-making process (*n* = 164; 83%, Supplementary Material). Despite the majority indicating direct involvement in EOL care in the professional subgroup analysis, nurses reported significantly less active participation in decision-making (64% nurses vs. 95% doctors, *p* < 0.001). Compared to the physicians, significantly fewer nurses reported to be involved in decision making (70% vs. 35%, *p* < 0.01), or in an EoL discussion (83% vs35%, *p* < 0.01) or to have initiated one (85% vs. 48%, *p* < 0.01). Figures 5, 6 show the involvement in EoL decisions of doctors and nurses/AHP.

**Figure 7:**
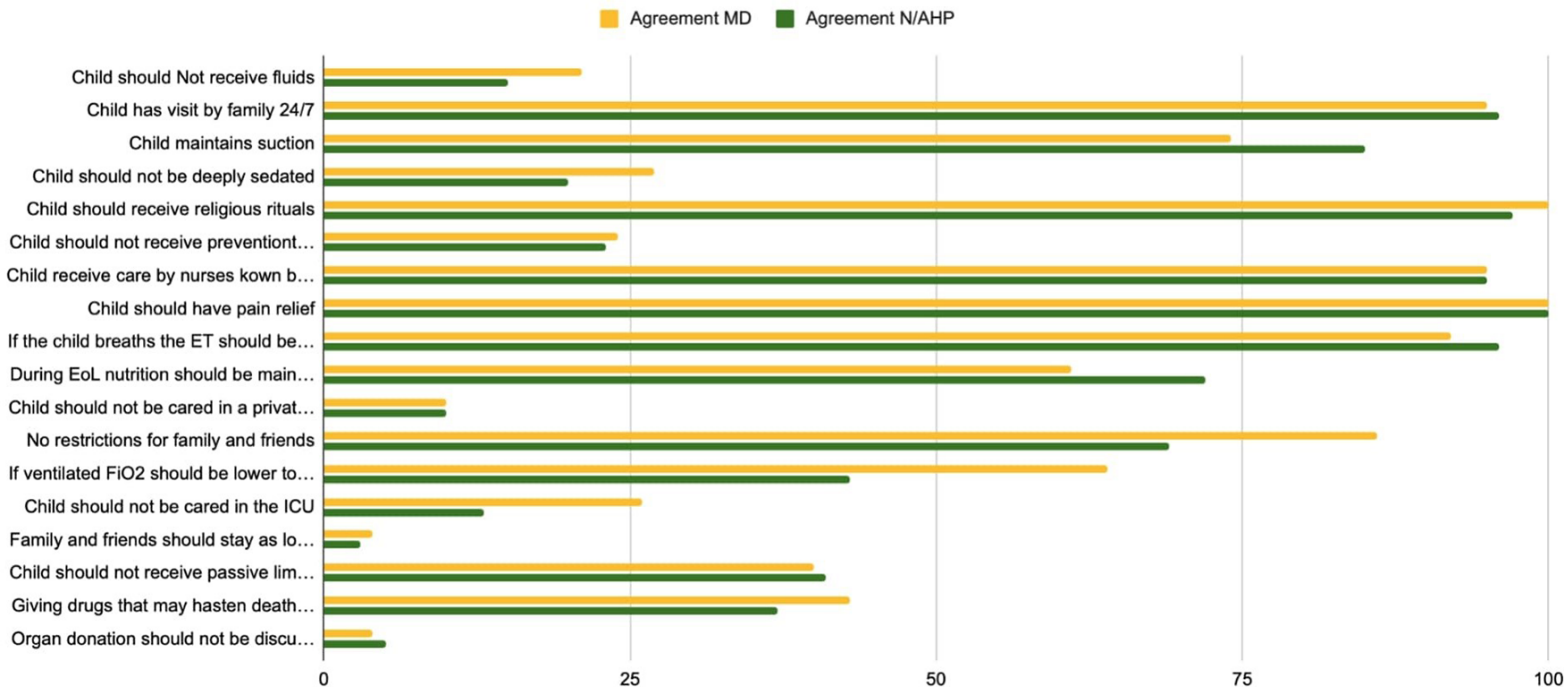
Questions 31–48. End of Life (EoL) practices.

In the final section of the questionnaire dealing with EOL in practice, attitudes and experiences in EOL care were similar across all PIC professionals: quality of life (QoL) remains the primary focus with the involvement of the family and the child in the decision-making process (Figure 7). Most respondents (*n* = 179; 90%, Supplementary Material) supported discussing organ donation with parents during EOL. There was a division of views about maintaining children under deep sedation for EOL care, and equal numbers contested the continuation of nutritional support at EOL (Supplementary Material S1).

In the sub-analysis by region, there was a significant difference (*p* < 0.05) between the three areas regarding the first question of the first section (Supplementary Material S2). There were different distributions among the three regions regarding the proper timing of EOL decision-making. In northern and southern countries, the timing was considered optimal compared to central Europe, where the majority of respondents believed decisions were taken too late (Supplementary Material S1). Our study also highlighted more frequent involvement of ethics committees in central Europe, and in Southern Europe a norm of deferring the final decision to parents regarding whether to stop life-sustaining therapy.

## Discussion

This study presents an overview of European children's critical care professionals' attitudes and perspectives about EOL care and EOL decision-making.

In European PIC, most deaths occur with a decision made about limitation of life-sustaining therapies, whether to stop resuscitation, not provide ECMO or to set a limit on inotropic support (4, 6, 11). Despite their standard ethical equivalence (29), our study highlights how PIC professionals consider not initiating an intervention and discontinuing it later as fundamentally different, and they perceive this as an important ethical issue. Whether this difference in views about the withholding and withdrawal of life-sustaining therapies more represents an emotional reaction to recurrently facing difficult decisions or is a valid location-specific moral norm is unknown. We found this more prevalent in nurses, arguably the best-placed professional group to assess the burden and benefits of ongoing life-sustaining technology at EOL. Therefore, they are perhaps the group most vulnerable to harm caused by having to continue to deliver invasive treatment that they disagree with.

The discrepancy in ethical views expressed by different professional members of the same speciality is of interest and potentially crucial given the difficulties in delivering good quality EOL care that inter-professional disagreement creates (25). Considering withholding treatment more ethically acceptable than withdrawal perhaps reflects experiential differences between bedside nurses and physicians. Ethical areas such as this could be explored in multi-professional meetings and be the subject of team-based educational programs. Formal training in moral dilemmas, followed by the opportunity to have experiential training, such as high-fidelity simulation, can provide teams with greater competence and confidence in this area (30–32). The lack of such training has been recognised as a serious deficiency in current medical education in Europe (30). Interventions to support and improve PIC teams' management of situations in which families and HCPs decide about the limitation of life-sustaining therapies for children are welcome (22). When a treatment no longer helps achieve the patient's care goals or cannot restore the minimum desired quality of life, withdrawing it can be in the child's best interests. But, looking after children in this situation can be very stressful for HCPs, especially those who do not clearly understand the moral arguments and relevant law (32).

The involvement of nursing and AHPs in EOL decision-making emerged as a critical issue for both nurses and physicians in our study. Beckstrand et al. published a report discussing physician and nurse-related barriers to delivering high-quality EOL care in the critical care context (25). Poor physician communication was the main obstacle that American adult critical care nurses reported, followed by physicians giving false hope (31). Increased workload and inadequate staffing were the predominant barriers to nurse participation in EOL discussions, compounded by insufficient education in EOL care (31). The value of interprofessional care and teamwork in the intensive care unit is well recognised (33). It refers to the advantage of care provided by a team of different HCPs with overlapping expertise in improving patient-oriented outcomes. A previous report regarding collaborative practice in clinical EOL discussions suggested that using formal guidelines, clear EOL collaborative decision-making processes, organisational support, and the provision of multi-professional education pathways may increase nurses' participation in EOL decisions (34).

We examined another crucial aspect of the decision-making process: timing. A recent report by Schorr et al. analysing the integration of palliative care into adult ICU highlighted that access to early palliative care consultation was associated with earlier EOL discussions (35). Local resources and realities vary from a clear separation of services to a proper integration of competencies, often in the same PIC HCPs (33–35). There is evidence from one centre that integrating a palliative care consultation service may be associated with an increased willingness to accept the withdrawal of life-sustaining treatment (9). Arguably, in other settings, given the prevalence of children with life-limiting conditions in PIC, palliative care involvement might increase admission rates in this cohort.

We analysed items related to EOL practices that suggested differences between European regions. Several studies have demonstrated that EOL care practices and preferences vary across countries in adult ICUs (36–38). Furthermore, cultural differences underlie people's attitudes and coping strategies when facing health, illness and death. Therefore, a grounded cultural analysis considering the role of family, religious background, the context of social relations, needs, values, interests and power positions of the different actors in EOL processes should be taken into consideration and might help explain how EOL care-related practices vary across regions (38).

The main limitations of our study relate to sample size and the recruitment strategy (ESPNIC members), which lead to more feedback from three main Western North, Central and South European countries, potentially leading to selection bias. For this reason, our results may not be fully representative of other European countries with potentially different EOL cultures and attitudes. Specifically, cultural differences based on Eastern orthodox faith, financial limitations and prior membership of the communist system are worth investigation. In addition, the questionnaire structure did not allow for qualitative responses, though we plan to perform further research using this to target less represented countries explicitly. Finally, missing data and unanswered questions lead to a potential loss of information.

## Conclusion

The EVOLvE study has shown that paediatric intensive care professionals' attitudes and experiences in EOL care are similar within each region, but differences in EOL decision-making in PIC persist between European regions. We also found that paediatric critical care nurses are less involved in EOL decision-making than physicians. Further research is needed to identify the cultural, religious, legal and resource differences underlying EOL decision-making and clinical practice discrepancies. We consider this proposed research and bespoke multi-professional ethics education to be the two essential steps to improve EOL care for children in European PIC.

## Data Availability

The raw data supporting the conclusions of this article will be made available by the authors, without undue reservation.
