# Supplementary material for: End-of-life decisions and practices as viewed by health professionals in pediatric critical care: A European survey study
Source: Front Pediatr. 2023 Jan 10;10:1067860. doi: 10.3389/fped.2022.1067860 (PMC9872024; doi:10.3389/fped.2022.1067860)

Supplementary Material

1. Supplementary Data

**The distribution of the answers for all the 48 items**

**ATTITUDES TOWARDS END-OF-LIFE CARE**

**Questions 1-3: Statements about withholding and withdrawing life supporting treatments**

| **Statements about withholding and withdrawing life supporting treatments** | **Strongly disagree** | **Disagree** | **Do not know** | **Agree** | **Strongly agree** |
| --- | --- | --- | --- | --- | --- |
| Withholding and withdrawing life support treatment are ethically the same | 23 (12%) | 66 (33%) | 15 (8%) | 60 (30%) | 34 (17%) |
| Withholding **OR** withdrawing life support treatment is unethical | 145 (73%) | 41 (21%) | 7 (4%) | 5 (3%) | 0 (0%) |
| Withholding life support treatment is more ethically acceptable than withdrawing life support treatment | 54 (27%) | 87 (49%) | 17 (9%) | 28 (14%) | 2 (1%) |

**Questions 4-16: How important are the following factors when making decisions about withholding or withdrawing life supporting treatment?**

| **How important are the following factors when making decisions about withholding or withdrawing life supporting treatment?** | **Not important at all** | **Quite important** | **Not sure** | **Important** | **Very**  **important** |
| --- | --- | --- | --- | --- | --- |
| Expected quality of life as viewed by the child | 0 (0%) | 3 (2%) | 9 (5%) | 54 (27%) | 132 (67%) |
| Expected quality of life as viewed by the parents | 2 (1%) | 14 (7%) | 8 (4%) | 102 (52%) | 72 (36%) |
| Expected quality of life as viewed by nursing colleagues | 10 (5%) | 35 (18%) | 22 (11%) | 101 (51%) | 30 (15%) |
| Expected quality of life as viewed by medical colleagues | 9 (5%) | 28 (14%) | 18 (9%) | 109 (55%) | 34 (17%) |
| Religious views of the child (if relevant) | 20 (10%) | 25 (13%) | 38 (20%) | 81 (41%) | 34 (17%) |
| Religious views of the parents | 26 (13%) | 41 (21%) | 25 (13%) | 79 (40%) | 27 (14%) |
| Religious views of the nursing team | 136 (69%) | 24 (12%) | 22 (11%) | 15 (7%) | 1 (1%) |
| Religious views of the medical team | 136 (69%) | 27 (14%) | 21 (11%) | 12 (6%) | 2 (1%) |
| The medical/nursing team has decided that the patient is unlikely to survive, even with ICU treatment | 2 (1%) | 11 (6%) | 14 (8%) | 77 (39%) | 94 (47%) |
| The medical/nursing team has decided that if the patient does survive, his/her neurological outcome is expected to be very poor | 4 (2%) | 10 (5%) | 12 (6%) | 81 (41%) | 91 (46%) |
| The medical/nursing team has decided that if the patient does survive, his/her quality of life is expected to be very poor | 5 (3%) | 14 (7%) | 15 (8%) | 82 (41%) | 82 (41%) |
| Fear of litigation or breaking the Law | 32 (16%) | 50 (25%) | 38 (19%) | 62 (31%) | 16(8%) |
| The ICU bed is needed for another critically ill patient | 143 (72%) | 25 (13%) | 19 (10%) | 10 (5%) | 1 (1%) |

**INVOLVEMENT IN END-OF-LIFE CARE DECISION**

**Questions 17-18**

|  | **Yes** | **No** |
| --- | --- | --- |
| Have you ever been directly involved in the care of a child where treatment has been withheld or withdrawn? | 191 (97%) | 7 (3%) |
| Have you ever been actively involved in the decision-making process to withhold or withdraw treatment in a child? | 164 (83%) | 34 (17%) |

**Questions 19-30**

|  | **Strongly disagree** | **Disagree** | **Do not know** | **Agree** | **Strongly agree** |
| --- | --- | --- | --- | --- | --- |
| In my experience, the timing of the discussion about the decision to withhold or withdraw life support treatment of a child is often too early. | 72 (36%) | 103 (52%) | 11 (6%) | 11 (6%) | 1 (1%) |
| In my experience, the timing of the discussion about the decision to withhold or withdraw treatment of a child is often just right. | 10 (5%) | 95 (48%) | 24 (13%) | 66 (33%) | 3 (2%) |
| In my experience, the timing of the discussion about the decision to withhold or withdraw treatment of a child is often too late. | 6 (3%) | 32 (16%) | 20 (10%) | 106 (54%) | 34 (17%) |
| I am always asked by my medical colleagues to participate in the decision-making process regarding end-of-life care of a child. | 12 (6%) | 43 (22%) | 30 (15%) | 77 (39%) | 36 (18%) |
| In my experience, I have always been actively involved in discussions with doctors about end-of-life decisions. | 12 (6%) | 44 (22%) | 15 (8%) | 9 (46%) | 35 (18%) |
| I have often initiated discussion with doctors about end-of-life care of a child. | 9 (5%) | 27 (14%) | 22 (12%) | 106 (54%) | 34 (17%) |
| In my experience, the child and/or their parents are always involved in discussions about end-of-life care of a child. | 1 (1%) | 26 (13%) | 12 (7%) | 99 (50%) | 60 (30%) |
| I think the child and/or their parents always need to be fully consulted before a decision to withhold or withdraw treatment of a child is made. | 2 (1%) | 9 (5%) | 10 (5%) | 66 (33%) | 111 (56%) |
| Involvement in ethical decision-making of issues such as withholding or withdrawal of treatment positively influences my job satisfaction. | 8 (4%) | 17 (8%) | 33 (17%) | 97 (49%) | 43 (22%) |
| In my unit is possible to have an involvement of the local ethical committee to help the decision making process | 23 (12%) | 19 (10%) | 35 (18%) | 79 (39%) | 42 (21%) |
| In my unit we regularly involved the local ethical committee to help the decision making process | 38 (19%) | 69 (35%) | 42 (21%) | 36 (18%) | 13 (7%) |
| Parents should have the final say wether to stop life support treatment for their life | 29 (15%) | 94 (47%) | 22 (12%) | 46 (23%) | 7 (4%) |

**END-OF-LIFE CARE IN PRACTICE**

**Questions 31-48**

|  | **Strongly disagree** | **Disagree** | **Do not know** | **Agree** | **Strongly agree** |
| --- | --- | --- | --- | --- | --- |
| During end-of-life care, the child SHOULD NOT continue to receive fluids to maintain hydration | 43 (22%) | 88 (44%) | 36 (18%) | 28 (14%) | 3 (2%) |
| The family and friends of the child SHOULD be permitted to visit at any time, day or night | 2 (1%) | 7 (4%) | 2 (1%) | 35 (18%) | 149 (75%) |
| During end-of-life care, oro/endotracheal suction SHOULD be continued to maintain the airway of the child | 6 (3%) | 29 (15%) | 27 (14%) | 82 (41%) | 50 (25%) |
| The child SHOULD NOT be kept deeply sedated | 46 (23%) | 72 (36%) | 38 (19%) | 36 (18%) | 2 (1%) |
| The child SHOULD always be given the opportunity to receive last rituals that are appropriate to the religious and spiritual beliefs of the child and their parents. | 1 (1%) | 1 (1%) | 0 (0%) | 36 (18%) | 155 (78%) |
| The child SHOULD NOT continue to receive all interventions to prevent pressure sores | 76 (38%) | 60 (30%) | 16 (8%) | 30 (15%) | 12 (6%) |
| The child SHOULD continue to receive care from nurses who know the child and family | 3 (2%) | 6 (3%) | 8 (4%) | 55 (28%) | 123 (62%) |
| The child SHOULD be provided with effective pain relief | 0 (0%) | 0 (0%) | 2 (1%) | 12 (6%) | 179 (90%) |
| If the child is able to breathe spontaneously, the endotracheal tube SHOULD be removed | 0 (0%) | 10 (5%) | 34 (18%) | 82 (41%) | 72 (36%) |
| During end-of-life care, the nutritional support of the child SHOULD be continued | 8 (4%) | 42 (21%) | 52 (26%) | 67 (34%) | 29 (15%) |
| The child SHOULD NOT be cared for in the privacy of a private room | 116 (59%) | 44 (22%) | 20 (10%) | 10 (5%) | 8 (4%) |
| The family and friends of the child SHOULD be permitted to visit the child at the bedside without a restriction on the number of family members and friends | 3 (2%) | 33 (17%) | 20 (11%) | 64 (32%) | 78 (39%) |
| If ventilated, the child’s inspired oxygen level SHOULD be reduced to 21% (air) | 16 (8%) | 43 (22%) | 57 (31%) | 56 (28%) | 22 (11%) |
| The child SHOULD NOT continue to receive care in the intensive care unit | 40 (20%) | 88 (44%) | 31 (18%) | 29 (15%) | 6 (3%) |
| The family and friends of the child SHOULD NOT be permitted to visit for as long as they want | 127 (64%) | 49 (25%) | 10 (6%) | 8 (4%) | 4 (2%) |
| The child SHOULD NOT continue to receive a full range of passive limb exercises | 36 (18%) | 48 (24%) | 57 (29%) | 48 (24%) | 9 (5%) |
| Giving drugs that may hasten death deliberately is acceptable | 37 (19%) | 59 (30%) | 35 (18%) | 53 (27%) | 14 (7%) |
| Organ donation should not be discussed as it may upset the family | 123 (62%) | 56 (28%) | 10 (6%) | 6(3%) | 3 (2%) |


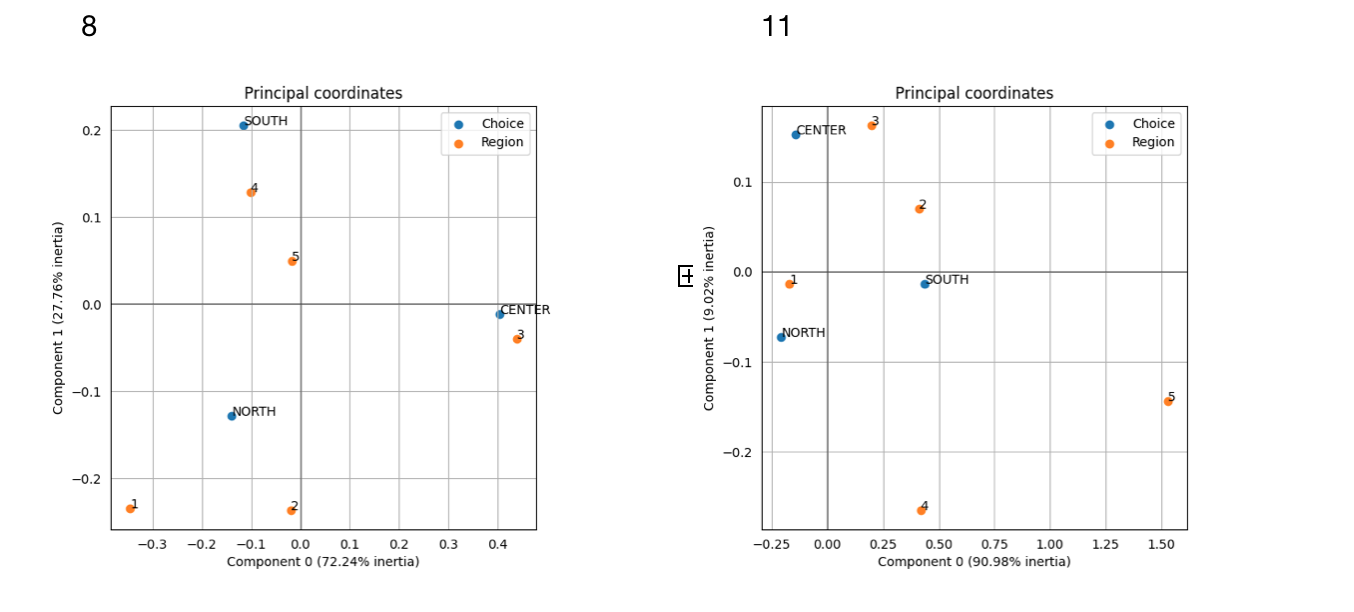

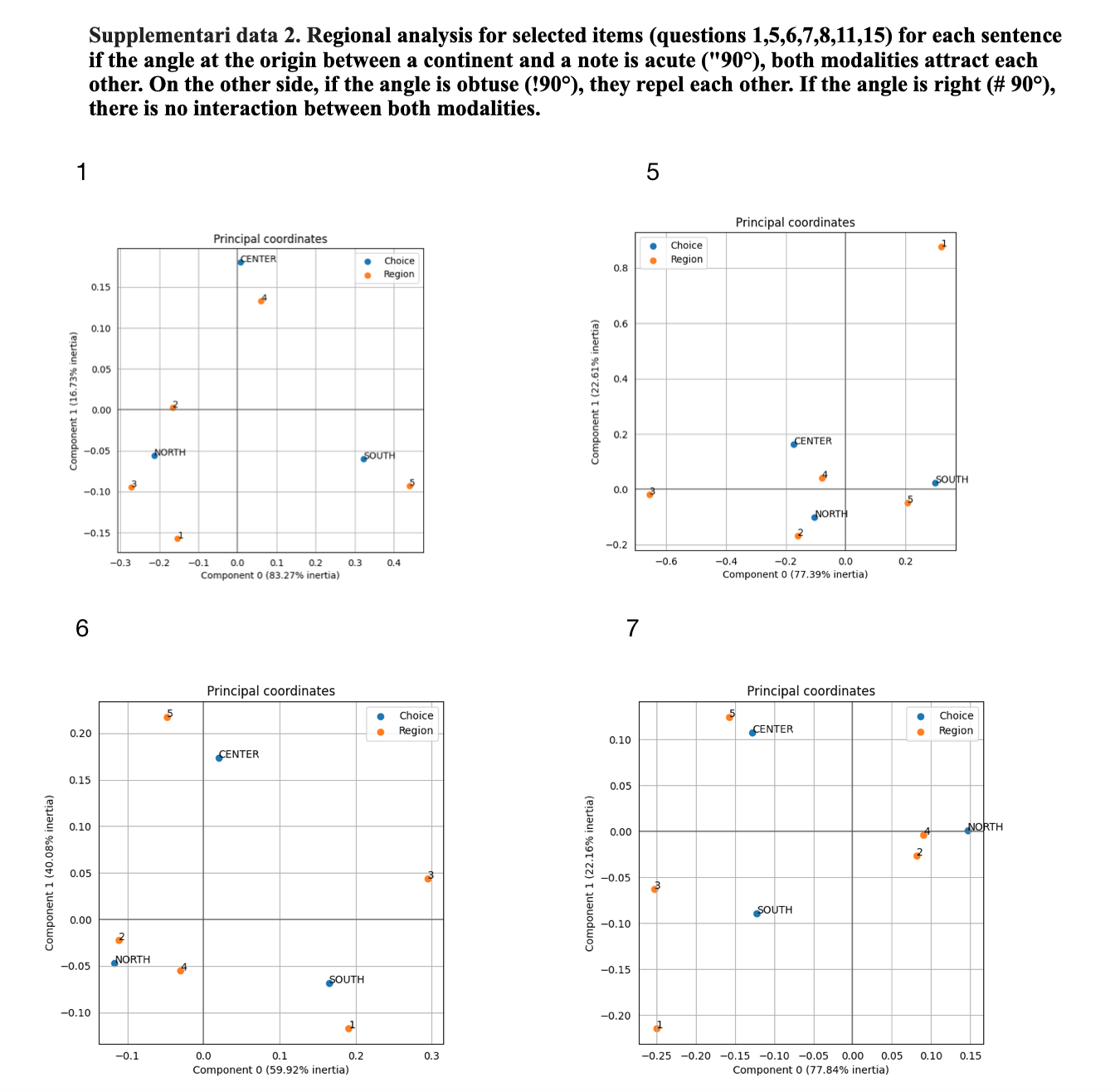


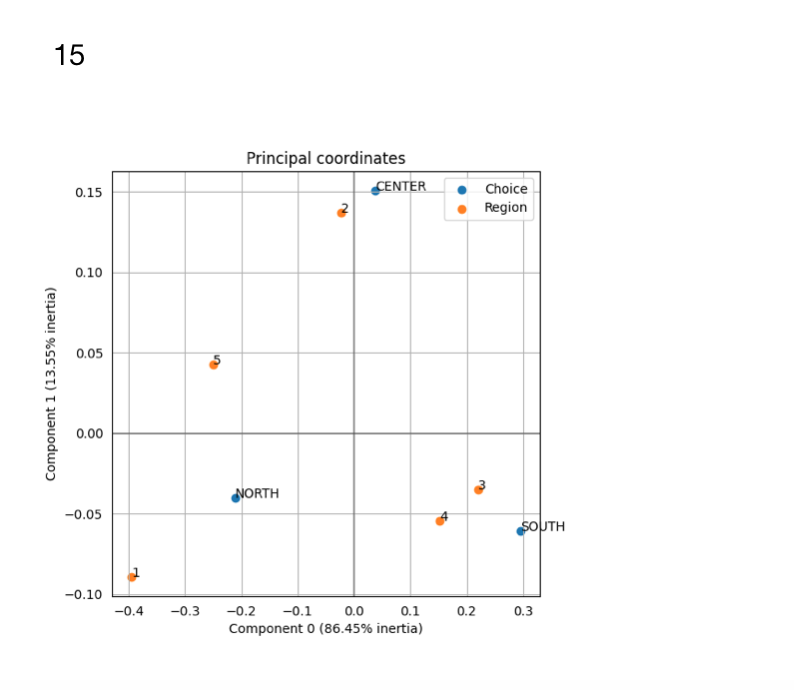

Supplement: Supplementary file 1 [file Datasheet1.docx]
